# **Optimization of single shot 3D breath-hold non-enhanced MR angiography of the renal arteries**

**DOI:** 10.1186/1532-429X-14-30

**Published:** 2012-05-19

**Authors:** Huan Tan, Ioannis Koktzoglou, Christopher Glielmi, Mauricio Galizia, Robert R Edelman

**Affiliations:** 1 Department of Radiology, NorthShore University HealthSystem, 2650 Ridge Avenue, Evanston, IL, 60201, USA; 2 The University of Chicago Pritzker School of Medicine, Chicago, IL, USA; 3 Siemens Healthcare, Cardiovascular MR R&D, Chicago, IL, USA; 4 Northwestern University Feinberg School of Medicine, Chicago, IL, USA

**Keywords:** Non-enhanced Renal MRA, 3D Steady-State Precession Acquisition, Breath-hold

## Abstract

**Background:**

Cardiac and navigator-gated, inversion-prepared non-enhanced magnetic resonance angiography techniques can accurately depict the renal arteries without the need for contrast administration. However, the scan time and effectiveness of navigator-gated techniques depend on the subject respiratory pattern, which at times results in excessively prolonged scan times or suboptimal image quality. A single-shot 3D magnetization-prepared steady-state free precession technique was implemented to allow the full extent of the renal arteries to be depicted within a single breath-hold.

**Methods:**

Technical optimization of the breath-hold technique was performed with fourteen healthy volunteers. An alternative magnetization preparation scheme was tested to maximize inflow signal. Quantitative and qualitative comparisons were made between the breath-hold technique and the clinically accepted navigator-gated technique in both volunteers and patients on a 1.5 T scanner.

**Results:**

The breath-hold technique provided an average of seven fold reduction in imaging time, without significant loss of image quality. Comparable single-to-noise and contrast-to-noise ratios of intra- and extra-renal arteries were found between the breath-hold and the navigator-gated techniques in volunteers. Furthermore, the breath-hold technique demonstrated good image quality for diagnostic purposes in a small number of patients in a pilot study.

**Conclusions:**

The single-shot, breath-hold technique offers an alternative to navigator-gated methods for non-enhanced renal magnetic resonance angiography. The initial results suggest a potential supplementary clinical role for the breath-hold technique in the evaluation of suspected renal artery diseases.

## Background

Renal artery stenosis (RAS) is an important cause of secondary hypertension and chronic renal failure [1]. Several imaging options are available, including CT angiography, MR angiography (MRA), radionuclide renography, and duplex sonography [2-5]. Contrast-enhanced (CE) MRA is commonly used for renal artery evaluation. However, recent studies [6,7] have found a strong association between nephrogenic systemic fibrosis and the use of gadolinium-based contrast material in patients with impaired renal function. Consequently, there is growing interest to use non-enhanced MRA techniques.

A cardiac and navigator-gated, magnetization-prepared 3D balanced steady-state free precession (Nav MP-SSFP) sequence is frequently used for non-enhanced MRA of the renal arteries [8-14]. The magnetization of the imaging region is first inverted, followed by a delay to allow fresh inflow of the arterial blood into the renal arteries prior to image acquisition. With the inherent dependence of the balanced SSFP readout on the T_2_/T_1_ ratio and the background suppression by magnetization preparation, the renal arteries appear bright compared to the surrounding tissues. A recent study found the technique to be 91% accurate for RAS in patients with chronic kidney disease [15]. However, in that study the patient’s breathing had to be precisely regulated to obtain reliable results, which may not be feasible in some patients. Moreover, the dependence of a navigator-gated free breathing acquisition on respiratory pattern can occasionally result in excessive scan times or blurring due to irregular breathing patterns.

A breath-hold acquisition could overcome the aforementioned limitations associated with the Nav MP-SSFP technique. A prior study by Maki and colleagues [9] reported a navigator-based SSFP technique outperformed a breath-hold SSFP in measurements of image quality and reader confidence for renal MRA. In Maki’s study, however, there was no mention of using advanced acquisition techniques such as parallel imaging, cardiac synchronization and partial Fourier encoding for the breath-hold technique. In addition, a signal-average of two was used to boost signal-to-noise ratio (SNR) which further limited the slice coverage. It was concluded breath-hold technique was not suitable for renal MRA.

In our study, we implemented a breath-hold, non-enhanced 3D SSFP technique (BH MP-SSFP) incorporating parallel imaging, cardiac synchronization (ECG gating) and partial Fourier encoding for renal MRA. The purpose of the study was to optimize the imaging parameters for BH MP-SSFP, and to evaluate the image quality in comparison with Nav MP-SSFP in volunteers and patients. Furthermore, an alternative magnetization preparation scheme for both BH and Nav SSFP was also investigated.

## Methods

### Breath-hold technique implementation

**Figure 1:**
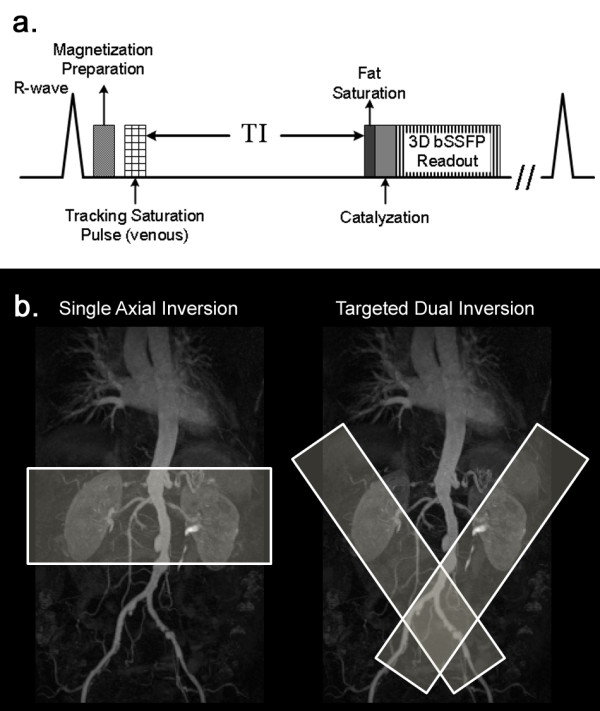
a) BH MP-SSFP pulse sequence diagram. b) The schematics of the magnetization preparation schemes: single axial inversion (left) and targeted dual inversion (right).

The BH MP-SSFP technique consists of a 3D balanced SSFP acquisition sequence, partial Fourier k-space trajectory with a reverse linear view order, single-shot acquisition along the phase-encoding direction, parallel imaging, slab-selective magnetization preparation pulse for background suppression, and cardiac triggering to ensure inflow during systole and data acquisition during diastole. The reverse linear partial Fourier acquisition starts within the half of k-space that is partially sampled, and traverses the remaining k-space linearly through the k-space origin to the highest frequency in the fully sampled half. A saturation RF pulse is applied caudally to the imaging plane to suppress venous inflow. Data acquisition is delayed by an inversion time (TI) to allow unsuppressed blood to enter the imaging region. A chemical-shift selective fat suppression pulse is applied prior to each 3D SSFP echo train, followed by an RF catalyzation sequence with linearly increasing flip angles to force the magnetization to approach steady state. The pulse sequence diagram of BH MP-SSFP is illustrated in Figure 1a.

### MR experiments

A total of fourteen healthy volunteers (ages 20 – 56, 10 male and 4 female) and nine patients (ages 63 – 90, 5 male and 4 female) were recruited for this study with approval from our Institutional Review Board. Written informed consent was received from each subject. Imaging was performed on a 32-channel 1.5 T scanner (MAGNETOM Avanto, Siemens Healthcare, Erlangen, Germany) equipped with high performance gradient coils (45 mT/m maximum gradient strength, 200 mT/m/ms slew rate). The body coil was used as the transmitter, and spine array (4 channels) and body array coils were used as the receiver. Subjects were positioned feet first and all imaging was performed at magnet isocenter.

Three experiments were conducted to evaluate the performance of BH MP-SSFP. Experiment 1 was designed to optimize imaging parameters for BH MP-SSFP using a single axial inversion magnetization preparation. Experiment 2 was designed to investigate the use of targeted dual inversion pulses in an oblique sagittal orientation as the magnetization preparation technique [16]. In the last experiment, the effectiveness of BH MP-SSFP was evaluated in patients.

#### *Experiment 1: Technical optimization*

In order to determine the optimal protocol for BH MP-SSFP, the following imaging parameters were varied: (a) TI, (b) acquisition excitation flip angle, (c) cardiac synchronization (ECG gating), and (d) magnetization preparation. The minimum and maximum allowable TI values were used to explore the dependency on arterial inflow. Scans were acquired using excitation flip angles of 50°, 75° and 90°, with and without ECG gating, and using inversion or saturation pulses for background suppression. The optimal parameters determined from this experiment were used in experiment 2 and 3.

Eight healthy volunteers participated in this experiment. The inter-shot interval for BH MP-SSFP was determined by the heart rate, and the following acquisition parameters were used: 75% asymmetrical echo readout, TR/echo time = 3.3 ms/1.4 ms, field of view = 450 × 248 mm, acquisition matrix = 400 × 220, 5/8 partial Fourier encoding sampling along the phase encoding direction, slab thickness = 52.8 cm, 6/8 partial Fourier sampling along the slab direction with 22 encodings yield an isotropic reconstructed resolution of 1.1 × 1.1 × 1.1 mm^3^, a reverse linear view ordering was used along both partial Fourier directions, receiver bandwidth = 781 Hz/pixel, partial Fourier encodings were reconstructed by zero filling. Generalized auto calibrating partially parallel acquisition (GRAPPA) [17] was applied along the phase encoding direction with 24 autocalibration lines. The resultant net acceleration was 1.8.

#### *Experiment 2: Targeted dual inversion preparation*

Conventionally, a single inversion slab was applied axially for background suppression, which also partially suppressed signal from inflowing blood due to its intersection with the aorta. Hence, BH MP-SSFP relied on a long TI to maximize inflow effect to maintain adequate arterial signal. Alternatively, rather than using a single inversion band, two inversion bands were placed in an oblique sagittal orientation to cover the mid and distal renal artery on both sides of the aorta [16]. Illustrated in Figure 1b, the new targeted dual inversion preparation scheme now avoids suppressing the blood signal in the aorta and extra-renal arteries, thus allowing more unsuppressed blood to reach distal arteries within a shorter time.

This new preparation scheme was tested in seven healthy volunteers with both BH and Nav MP-SSFP techniques. Nav MP-SSFP MRA images were acquired using the commercially available navigator-gated NATIVE TrueFISP sequence (Siemens Healthcare, Erlangen, Germany). Imaging parameters were similar for both techniques except for the following differences: 1) BH MP-SSFP used a single-shot readout for the in-plane phase-encoding direction, and Nav MP-SSFP used a three-shot readout without partial Fourier encoding; 2) BH MP-SSFP employed a center-to-out partial Fourier trajectory along the slab encoding direction, whereas Nav MP-SSFP used an out-to-center partial Fourier trajectory. Navigator acceptance window for Nav MP-SSFP was 10 mm. A crossed-pair navigator was placed at the dome of the right hemidiaphragm to synchronize data readout to minimize respiratory motion. Three TI values, maximum allowable TI (TI_max_, determined by the R-R interval and the echo train length within each R-R interval of BH MP-SSFP), TI_max_ – 100 ms, TI_max_–200 ms, were used for both techniques to evaluate the inflow dependency. TI_max_ in our volunteer study ranged from 450 to 685 ms. The remaining imaging parameters were the same as for Experiment 1.

#### *Experiment 3: Patient study*

Nine patients with a history of peripheral artery disease and hypertension were enrolled to test the clinical feasibility of BH MP-SSFP. The imaging parameters for BH and Nav MP-SSFP were identical to Experiment 2 with TI_max_ = 659 ± 133 ms. The number of encodings along the slab direction varied from 32–48 according to the individual heart rate to maintain an average breath-hold time of approximately 20 seconds. In two patients, Nav MP-SSFP scan was prescribed with the standard clinical protocol: TR/echo time/flip angle = 3.9 ms/1.7 ms/90°, TI = 620 ms, segment TR = 700 ms, field of view = 360 × 268 mm, slab thickness = 96 mm, resolution = 1.3 × 1.2 × 0.9 mm^3^, bandwidth = 783 Hz/pixel, GRAPPA factor = 2, no partial Fourier encoding along the in-plane phase encoding direction, 6/8 partial Fourier encoding along the slab encoding direction, navigator acceptance window = 10 mm. Full k-space was acquired in three shots. CE-MRA was acquired in seven out of nine patients after the non-enhanced sequences. The image quality was assessed visually by a radiologist with more than ten years experience interpreting body MRA to determine the diagnostic quality.

### Data analysis

Quantitative analysis was completed in healthy volunteers by calculating the image signal-to-noise ratio (SNR) and contrast-to-noise ratio (CNR). Signal intensities (SI) were measured in user-specified regions of interests (ROI) that were placed in the intra- and extra-renal arteries (IRA and ERA), kidney parenchyma (KP), soft tissue surrounding the ERA (ST), and nearby paravertebral muscle (PM). The ROIs were of similar size and position between BH and Nav MP-SSFP. SNR and CNR were calculated based on the ROIs. SNR of the renal arteries were calculated as SNR_IRA/ERA_ = SI_IRA/ERA_/STD_PM_ where STD stands for the standard deviation. The standard deviation of nearby PM was used here to estimate the noise in place of background air to minimize the possible biases introduced by parallel imaging [18]. CNR was calculated as CNR_ERA, ST_ = (SI_ERA_ – SI_ST_)/STD_PM_, CNR_IRA, KP_ = (SI_IRA_ – SI_KP_)/STD_PM_.

Qualitative comparison of image quality obtained with BH and Nav MP-SSFP was performed using stacks of axial and coronal thin maximum intensity projection (MIP) images (20 mm thick with 5 mm overlap) derived from Experiment 2. Only images with TI_max_ were used in the qualitative review. The diagnostic quality of the images (extra-renal and intra-renal branch visualization for left and right sides) was evaluated by a radiologist with over ten years of experience blinded to the sequence type using a Likert scale of 0–4: 0, non-diagnostic; 1, poor quality and observer not confident; 2, fair quality and observer marginally confident; 3, good quality and observer confident; and 4, excellent quality and observer highly confident.

Statistical differences in SNR and CNR were identified using non-parametric Friedman tests. Wilcoxon signed-rank tests were used to identify differences in the qualitative image quality scores. A P value less than 0.05 was considered to indicate the presence of a significant difference.

## Results

### Experiment 1: Optimization of imaging parameters

**Figure 2:**
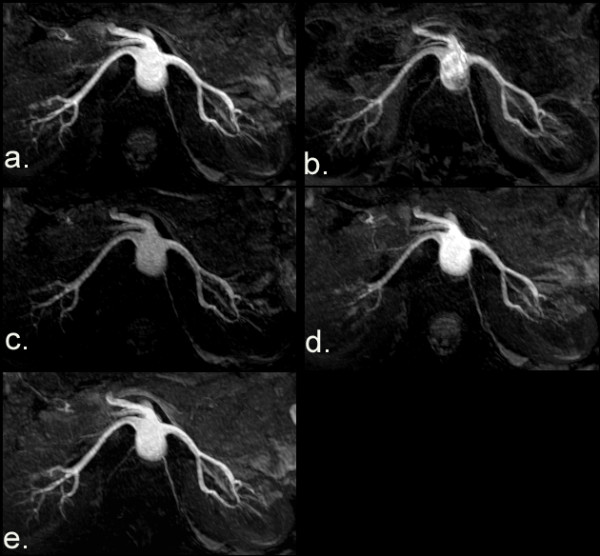
**MRA MIPs from BH MP-SSFP parameter optimization**. The images were obtained using the following imaging parameters: **a**) using optimal parameters consisting of an excitation flip angle = 90°, inversion preparation, maximum allowable TI, and ECG gating; **b**) using the minimum TI; **c**) using an excitation flip angle = 50°; **d**) without ECG gating; and **e**) using a saturation preparation pulse.

The optimal parameters for BH MP-SSFP were determined via Experiment 1 (Figure 2). The highest SNR and CNR were obtained with the use of a 90° flip angle for the True-FISP readout train and ECG gating. The visualization of distal portions of the renal arteries was optimized when TI_max_ was used. The SNR and CNR of BH MP-SSFP images with TI_max_, 90° excitation flip angle and ECG gating were significantly higher (P < 0.05) than the ones acquired separately with minimum TI, lower flip angles, and no ECG gating. There were no significant differences in SNR and CNR between BH MP-SSFP images acquired with inversion and saturation preparation pulses (*P* > 0.05) when the single axial preparation scheme was used.

### Experiment 2: Inversion slab configuration

**Figure 3:**
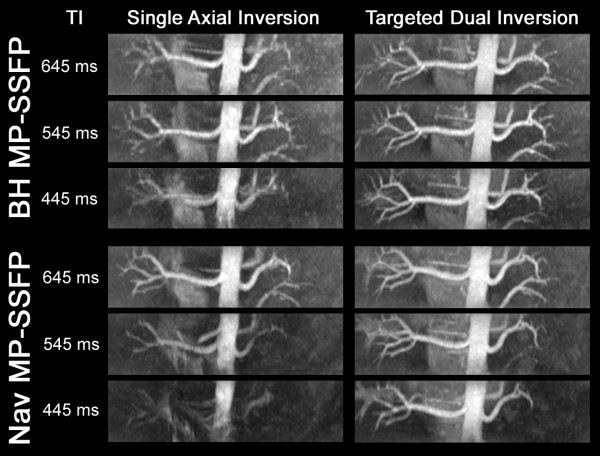
MRA MIP Comparisons between single axial inversion and targeted dual inversion with different TI values.

In this experiment, the average scan time was 20 seconds for BH MP-SSFP and 145 seconds for Nav MP-SSFP. With the single axial inversion preparation, the image quality of the renal arteries was found to be dependent on TI. However, this dependence was substantially diminished when the targeted dual inversion preparation was used, as illustrated in Figure 3. Significantly improved SNR and CNR within intra-renal arteries were obtained with the targeted dual inversion for all TIs (*P* < 0.05).

**Figure 4:**
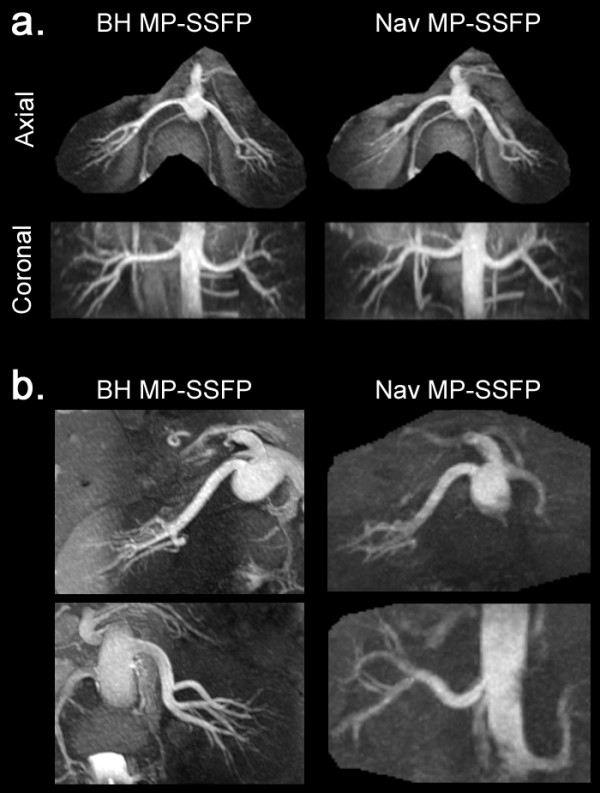
**Comparison of MRA MIPs obtained with BH and Nav MP-SSFP from two healthy volunteers**. **a**) Normal acquisition from volunteer 1 where the image parameters for BH and Nav MP-SSFP were matched. **b**) MRA images from volunteer 2 when contralateral arteries were located at substantially different levels. The BH MP-SSFP images were obtained in two separate breath-holds for the left and right renal arteries (24 seconds each side). The Nav MP-SSFP images were obtained using a single think slab (96 mm, 7 minutes and 26 seconds acquisition time). The suboptimal visualization of the left renal artery on the Nav MP-SSFP was a consequence of fewer unsaturated spins reaching the caudally located left renal artery as compared with the right renal artery, within the inflow time specified by the TI.

**Figure 5:**
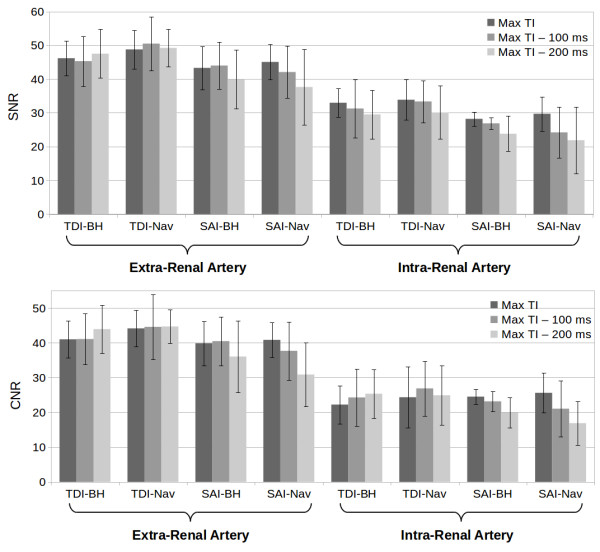
SNR and CNR results for BH and Nav MP-SSFP with both magnetization preparation schemes (TDI – Targeted Dual Inversion, SAI – Single Axial Inversion).

Both BH and Nav MP-SSFP demonstrated excellent image quality in healthy volunteers (Figure 4). In subjects whose contralateral arteries were located at substantially different levels, separate breath-hold scans were prescribed for each side (Figure 4b). There was no significant difference (*P* > 0.05) in SNR and CNR between BH and Nav MP-SSFP for comparable TI and inversion slab configurations. The SNR and CNR results are shown in Figure 5.

The mean and median of the image quality scores are reported in Table 1. No significant difference was found between Nav and BH MP-SSFP in all segments (*P* > 0.05) with the same magnetization preparation scheme. The image quality score for the left intra-renal arteries was significantly better with the dual targeted inversion (*P* < 0.05), while differences for the rest of the segments between the two preparation schemes were not significant.

**Table 1:** Qualitative image quality scores

	**Extra-renal Artery**		**Intra-renal Artery**	
**Median**	**Left**	**Right**	**Left**	**Right**
**BH MP-SSFP**	4	4	4	4
**Nav MP-SSFP**	4	4	4	4
	**Extra-renal Artery**		**Intra-renal Artery**	
**Mean ± S.D.**	**Left**	**Right**	**Left**	**Right**
**BH MP-SSFP**	3.88 ± 0.33	3.92 ± 0.27	3.31 ± 0.93	3.54 ± 0.81
**Nav MP-SSFP**	4.00 ± 0.00	3.93 ± 0.26	3.50 ± 0.84	3.61 ± 0.74

### Experiment 3: Patient imaging

**Figure 6:**
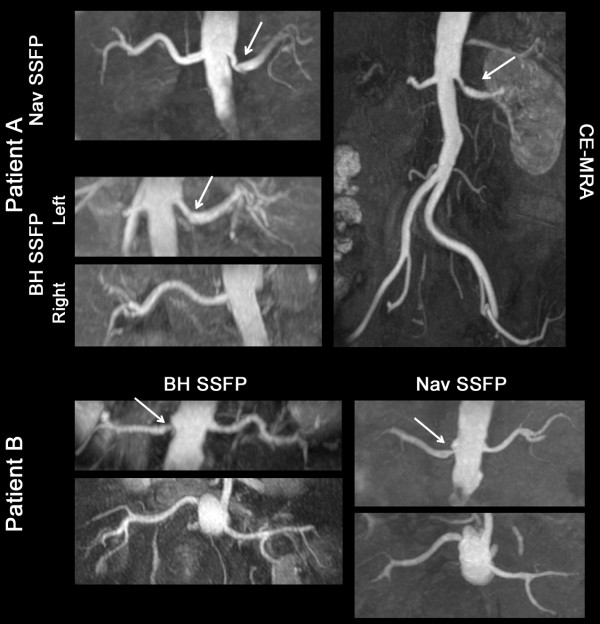
**MRA MIPs of two patients with renal stenoses (indicated by the arrow). Nav MP-SSFP was acquired with the clinical protocol**. The slab coverage of Nav MP-SSFP was twice as thick as MP-BH SSFP. The scan time for BH MP-SSFP was 21 seconds for both patient A and B. The scan times for Nav MP-SSFP were 373 and 588 seconds for patient A and B, respectively.

Nav MP-SSFP was not acquired in two patients because of scan failure due to irregular breathing pattern. The average scan time was 20 seconds for BH MP-SSFP, 208 seconds for Nav MP-SSFP with matching imaging parameters and 451 seconds with the clinical protocol. Four patients were found to have moderate to severe RAS. Both BH and Nav MP-SSFP demonstrated good diagnostic image quality based on visual assessment. The location and severity of the RAS, as confirmed by the corresponding CE-MRA, were correctly identified by BH and Nav MP-SSFP (Figure 6). Slightly better image quality in intra-renal arteries was observed with Nav MP-SSFP. Overall, BH MP-SSFP showed good agreement with Nav MP-SSFP in patients with regards to the depiction of local stenoses while achieving a marked reduction in scan time.

## Discussion

The purpose of this study was to develop a breath-hold alternative to navigator-gated non-enhanced renal MRA. We found that the breath-hold technique demonstrated comparable SNR and CNR to the navigator-based approach in healthy volunteers. A targeted dual inversion preparation minimized the dependency on inflow effect and significantly improved the image quality for the intra-renal arteries. In a pilot patient study, BH MP-SSFP provided comparable diagnostic information and accurately identified the presence of arterial stenoses, while providing a mean seven-fold reduction in scan time.

Our findings differ from the results reported by a previous study by Maki et al. [9]. In general, BH MP-SSFP generally provided comparable image quality to the navigator-based approach despite the substantial reduction in scan time. We believe the improved performance of the BH MP-SSFP in our study is the collective result of several technique optimizations, including the use of ECG triggering, parallel imaging, partial Fourier k-space trajectory with a reverse linear view ordering, single-shot data acquisition along the in-plane phase-encoding direction, and use of a targeted dual inversion preparation. ECG triggering ensures inflow occurred during systole and image acquisition occurred during diastole. The reverse partial Fourier trajectory and parallel imaging minimizes the number of RF pulses between the magnetization preparation and the center of k-space, which may reduce saturation effects. Also, the partial Fourier technique minimizes the time from the fat saturation pulse to the center of k-space, ensuring robust fat suppression. Finally, the targeted dual inversion preparation maximizes the inflow signal given a certain TI, thus optimizing arterial contrast.

Our commercially available implementation of Nav MP-SSFP (navigator-gated NATIVE TrueFISP, Siemens Healthcare, Erlangen, Germany) used a three-shot data acquisition to reduce the echo train length within each R-R interval in order to obtain adequate inflow of arterial spins and fat suppression. The gating efficiency, defined as percentage of acquired imaging data that falls within the navigator acceptance window, of the Nav MP-SSFP is typically on the order of 30%, whereas BH MP-SSFP is 100% efficient. The improved efficiency of the breath-hold technique arising from the combination of a single-shot acquisition and breath-holding helps explain why image quality is largely comparable to the navigator-based technique, despite the disparity in scan times.

Navigator-based non-enhanced renal MRA has proven to be robust and accurate; consequently, it is in widespread clinical use. As compared with Nav MP-SSFP, the short scan time of BH MP-SSFP provides increased flexibility with regard to using technical variations or repeating the sequence in case of suboptimal image quality. For instance, it may be helpful to reacquire the data using a different TI or orientation of the imaging slab. The breath-hold time for BH MP-SSFP ranged from 17–21 seconds in our patient study. For patients unable to hold their breath for this duration, scan time can be reduced by decreasing the number of slices at the expense of spatial coverage. The breath-hold technique may also be helpful in situations where the diaphragm is not within the field of view, thereby precluding use of navigator-based techniques.

Some drawbacks of BH MP-SSFP were apparent in this study. For instance, the use of partial Fourier encoding in the slab direction resulted in image blurring. The image sharpness in the slab encoding direction can be improved by using a full Fourier acquisition at the expense of less slab coverage. Alternatively, homodyne [19] or projections onto convex sets (POCS) reconstruction [20] could also be used to reduce the blurring. However, it is worth noting that since partial Fourier encoding was used in both phase and slab encoding directions, homodyne reconstruction can be processed with only one direction, while the other direction must use zero filling. Another drawback given the relatively thin slab used for BH MP-SSFP is that it may not be possible to image the right and left renal arteries within the same breath-hold, if the contralateral arteries are located at substantially different levels. In this circumstance, two separate breath-holds would be required. On the other hand, inflow-dependent saturation effects, which may occur for the more inferiorly positioned renal artery using a standard thick slab navigator-based approach, are avoided.

## Conclusions

In conclusion, an optimized breath-hold MRA technique demonstrated good diagnostic image quality while achieving a mean seven-fold reduction in scan time compared with a commercially available navigator-based technique. Despite minor drawbacks, the method has the potential to greatly reduce study time and may be of particular value for patients with inconsistent breathing patterns. Further study is warranted to determine its clinical accuracy for patients with renal artery disease.

## Abbreviations

RAS, Renal Artery Stenosis; MRA, Magnetic Resonance Angiography; MP, Magnetization-prepared; SSFP, Steady State Free Precession; Nav, Navigator-gated; BH, Breath-Hold; TI, Inversion Time; SNR, Signal-to-Noise Ratio; CNR, Contrast-to-Noise Ratio; ROI, Region of Interests; SI, Signal Intensities; IRA, Intra-Renal Arteries; ERA, Extra-Renal Arteries; KP, Kidney Parenchyma; ST, Soft Tissue surrounding ERA; PM, Paravertebral Muscle.

## Competing interests

CG is an employee of Siemens Medical Solutions USA, Inc.

## Authors’ contributions

HT, IK and RRE participated in the design and development of the pulse sequence, study design, data acquisition and analysis, manuscript drafting and figure preparation. CG participated in the design and development of the pulse sequence and study design. MG participated in the data analysis. All authors read, edited and approved the final manuscript.
